# Facts for Food Economists

**Published:** 1917-11-17

**Authors:** 


### THE LEAGUE OF NATIONAL SAFETY.

### Facts for Food Economists.

SIR ARTHUR YAPP, Director-General of National Economy, speaking at Harrods on Tuesday, November 6, on "Food Ways and Means in War-time," gave some interesting figures with regard to our urgent need for frugality in the use of sea-borne foodstuffs. In normal times 75-80 per cent. of all the grain used in this country came from abroad; five-sixths of all our cheese was imported, two-thirds of the butter, three-quarters of the meat, and all the tea, coffee, and cocoa. As the U-boats were still sinking ships faster than we were building them some very drastic means of reducing our consumption must be undertaken by us voluntarily or we should perforce be put upon a system of rationing. Such a system, he said, would mean the formation of yet another Government Department, and unless the good will of the people were enlisted it would be well-nigh impossible to detect fraud.

In dealing with the food position in Germany some interesting data were given indicating the shortage of all foodstuffs there. Before the war German imports amounted to 500,000,000 sterling, or £8 per head per annum. On the outbreak of hostilities nearly all of this had been cut off by our blockade, and the German now, instead of getting 4,460 calories per day, was reduced to 1,775, or in the case of a man doing hard manual work a little more. The rations allowed per worker per head were: Bread, 4¼ lb. per week; meat, 8¾ oz. per week; sugar, 6 oz. per week; butter, 3⅜ oz. per week; potatoes, 7 lb. per week; and 26 eggs a year. As a matter of fact there was a considerable surplus of food in Allied and neutral countries, and the difficulty lay in transporting it.

At the close of his address, Sir Arthur Yapp invited all present to join the League of National Safety, an organisation striving to do all in its power to check waste and reduce the consumption of food. Within three days of its inauguration the League had 13,000 members, and it was hoped by Christmas to enrol 10,000,000. To each member would be issued an anchor badge to wear in the dress or in the buttonhole. We learn that full time voluntary helpers are required for clerical work in connection with the scheme. Those willing to serve should communicate with Lady Hunter, Grosvenor House, W. 1.

Messrs. Harrods are giving a series of lectures in connection with the scheme on Tuesdays and Fridays at 11 A.M., followed by a practical demonstration. The subjects announced include the cooking of cereals, meat, fish, and fruit, and special dishes for children and invalids. The attendance at the first lecture was close upon 3,000, and, judging by the applause, Sir Arthur Yapp is justified in hoping that the ladies present will not allow their enthusiasm to wane before real reforms in the kitchen have been effected.

